# Study of aged central auditory function using the auditory middle latency response

**DOI:** 10.1016/j.clinsp.2023.100245

**Published:** 2023-07-19

**Authors:** Anna Caroline Silva De Oliveira, Yara Bagali Alcântara, Viviane Borim De Góes, Pedro de Lemos Menezes, Eduardo Federighi Baisi Chagas, Milena Sonsini Machado, Ana Claudia Figueiredo Frizzo

**Affiliations:** a Postgraduate Program, Faculty of Philosophy and Sciences (FFC), Universidade Estadual Paulista (UNESP), Marília, SP, Brazil; b Postgraduate at Program of the Northeast Network of Biotechnology (RENORBIO), Universidade Federal Rural de Pernambuco (UFRPE), Recife, PE, Brazil; c Program Research in Health, Centro Universitário CESMAC, Macéio, AL, Brazil; d Speech Language Pathology Department, Universidade Estadual de Ciências da Saúde de Alagoas (UNCISAL), Macéio, AL, Brazil; e Postgraduate Program in Structural and Functional Interactions in Rehabilitation, Universidade de Marília (UNIMAR), Marília, SP, Brazil; f Postgraduate Program, Faculdade de Medicina da Marília (FAMEMA), Marília, SP, Brazil; g Speech Language Pathology Department and Graduate Program in Speech Language Pathology, Faculdade de Filosofia e Ciências (FFC), Universidade Estadual Paulista (UNESP), Marília, SP, Brazil

**Keywords:** Auditory evoked potentials, Acoustic stimulation, Aged, Auditory cortex

## Abstract

**Objective:**

Investigate the auditory function of the elderly using the middle latency potentials.

**Methodology:**

Group 1 (G1): 20 healthy individuals of both genders, older than 60 years, without hearing loss. Group 2 (G2): 20 healthy individuals of both sexes, older than 60 years, with hearing loss in frequencies from 4 to 8 kHz. Potential recording was performed with unilateral and bilateral stimulation and the Binaural Interaction Component was calculated.

**Results:**

Na latency in C3A1 was greater in the stimulation of the right ear in G2 and the amplitude of Na-Pa was greater in the stimulation of the right ear and recording in C3A1 in G1. The latency of the Pa component was higher in the stimulation of the right ear recorded in C4A2. The Pb component in G2 by bilateral stimulation and recorded in C4A2 had higher latency. The first and second negative and positive peaks presented greater amplitude in G1. In C3A1, the 1^st^ negative peak was more negative in G1 and the 2^nd^ positive peak showed greater amplitude in C4A2 in both groups.

**Conclusion:**

The transmission of auditory information to the primary auditory cortex is impaired with aging, especially in unilateral stimulation, reinforced by losses in elderly people with peripheral hearing loss, such as in the binaural interaction at the cortical and subcortical levels. Thus, the AMLR has shown to be a sensitive examination to investigate neuroauditory disorders in the elderly, especially related to high-frequency hearing loss and primary auditory cortex dysfunctions caused by the aging process.

## Highlights

• Aging impairs binaural interaction in cortical and subcortical regions, elderly people with hearing loss show differences in responses between the ears/electrodes.
• Elderly people without hearing loss show differences in responses between the hemispheres.
• Binaural interaction is more impaired in older adults with high-frequency hearing loss.

## Introduction

Currently, the number of elderly people in Brazil is increasing due to the reduction in the birth rate and the increase in life expectancy.^1^ Consequently, this increase must be accompanied by the improvement or maintenance of health and, thus, offer the quality of life to this population.

It is noted that with aging, there may be difficulty in locating the sound source, in perceiving and understanding speech in noise, which are some of the binaural abilities. They demonstrate the importance of binaural integration, essential for communication and which may be compromised even in the absence of peripheral hearing loss.^2^

The Auditory Middle Latency Responses (AMLR) have been used in Audiology scientific research as a complementary method for assessing auditory function and binaural processing. Since this measure sensitizes the hearing assessment and brings more accurate information regarding the processing of the auditory information and provides critical information for audiologic rehabilitation strategies.^3^

Because the binaural processing presents greater activity after the brainstem, performing AMLR is appropriate as it is generated by structures at higher levels from the brainstem to the primary auditory cortex aged.^4^, ^5^, ^6^ In addition, the binaural in AMRL was shown to be sensitive to central auditory deficits, resistant to high-frequency hearing loss, and clinically applicable in this study population.^3^

Cortical auditory potentials examinations showed age-related changes in auditory evoked responses, with increased P1 and P2 latencies when diagnosed in young adults, which may be related to the synchrony of the cortex in the sensory prefrontal region, and a longer processing of sensory information.^7^ There was also an increase in latency (P1-N1-P2) in elderly people with hearing loss when comparing cortical responses in elderly people with and without age-related hearing loss.^8^

Due to the maturation process of the structures responsible for generating the AMLR, the amplitude of the components is highly related to age.^9^ Therefore, AMLR may vary according to age, with greater amplitude in children and smaller amplitude in the elderly, due to cell degeneration caused by aging.^10^

According to some studies, there is a worsening in the AMLR response in older individuals, and there is a strong correlation between the quality of the response and increasing age.^11^ The study by Tlumak^12^ observed a difference between the amplitudes of the AMLR when there is a difference of 40 years between the subjects' ages.

In this sense, this study seeks the response to the following questions: “Is there a change in the AMLR in the elderly?”; “Is there a difference in the AMLR when comparing the records of the two cerebral hemispheres in the elderly without hearing loss at high frequencies?”; “Are monaural and/or binaural abilities compromised regardless of the auditory threshold at high frequencies?”; “Is the process of binaural interaction impaired in the two groups of elderly?”.

Hearing and understanding speech require processing spoken words at the cortical level. Thus, the AMLR can be an important tool in the management of the specific demands of the elderly in relation to their communication difficulties, and in the establishment of more efficient auditory rehabilitation strategies.^9^ Thus, it is important to evaluate the central region of the elderly with and without a hearing loss for the differential diagnosis, since the perceptual understanding may be related to the hearing loss or to the involvement of the Central Nervous System (CNS).

In this context, the aim of the study was to investigate the auditory function of the elderly using the middle latency potentials, to analyze and compare the unilateral and bilateral stimulation in the elderly.

## Materials and methods

The study was approved by the Research Ethics Committee of the institution under number 1.905.689. A descriptive and analytical cross-sectional study that followed compliance according to the STROBE Declaration, included 40 subjects, sampled by convenience, divided into two groups:

• Group 1 (G1): 20 individuals of both sexes (15 female and 5 male), older than 60 years right-handedness and tone audibility threshold in the frequencies of 0.25, 0.50, 1, 2, 3, 4, 6 and 8 kHz ≤ 25 dBHL (symmetrical thresholds within 15 dB) and Speech Reception Threshold (SRT) ≤ 25 dBHL, according to Lloyd and Kaplan's classification (1978);
• Group 2 (G2): 20 individuals of both sexes (16 female and 4 male), older than 60 years of right-handedness and who had symmetric bilateral sensorineural hearing loss (symmetrical thresholds within 15 dB) with tonal audibility thresholds ranging from 30 at 70 dBNA, considering frequencies from 3 to 8 kHz, and frequencies from 0.25, 0.50, 1, 2 ≤ 25 dBNA, according to the classification by Lloyd and Kaplan (1978). The configuration of the audiometric curve was symmetrical in both ears, with a difference between the interaural audibility thresholds for the assessed frequency ≤ 15 dBHL and air-bone gap of up to 10 dB and Speech Reception Threshold (SRT) ≤ 25 dBHL.

The study did not include individuals outside the age range of the studied group, individuals who had any cognitive impairment diagnosed by a neurologist, and who did not present preserved functionality for activities of daily living, used ototoxic drugs, had a history of exposure to noise, smoking and changes in middle ear and/or hearing loss in the frequencies of 500 Hz, 1 kHz and 2 kHz and/or thresholds ≥ 71 dB HL in frequencies from 4 to 8 kHz.

The two-channel auditory evoked potential equipment Bio-logic Evoked Potential System (EP) (Seattle, WA/USA) and ERA-39 intra-aural earphones were used. The ASC II software (Seattle, WA/USA) was used to extract data from each record obtained for the calculation of the Binaural Interaction Component (BIC), which corresponds to the sum of the responses obtained with the unilateral stimulation subtracted from the response obtained with the bilateral stimulation through the grand-average of the recorded waves with unilateral and bilateral stimulation.

As a pre-collection procedure, the authors performed: audiological anamnesis, an inspection of the external acoustic meatus, and Mini-Mental State Examination to characterize the sample, with 30 as the cut-off score, according to the median scores proposed by Brucki et al., 2003 tympanometry, audiometry tonal threshold for characterization of samples and groups and Speech Reception Threshold (SRL).

For AMLR recording, the subjects were accommodated in an acoustically treated room and temperature of 24°C, positioned in a reclining chair and they all received the same guidelines for keeping relaxed, but alert, their eyes open trying to stare at a fixed spot somewhere in the room to avoid moving the head. This way, the records (in all the subjects) did not present more than 10% of artifacts from the total of 1000 stimuli.

The electrodes were fixed with microporous adhesive tape after the skin was cleaned with abrasive paste and positioned at C3 and C4 (left and right temporal-parietal junction) with reference to A1 and A2 (left and right ear lobe), and ground on the Forehead (F4). This arrangement assures the observation of the ipsi and contralateral measures and optimizes the recording of cortical activity. The impedance of each electrode did not exceed 5 Kohms and did not exceed 2 Kohms between the electrode impedances.^10^

For the AMLR recording, unilateral click-stimuli were used in the right ear and left, and then bilateral stimuli (simultaneously in both ears), rarefaction polarity at 30 dB NS, with a presentation rate of 11 stimuli/second, time of analysis (window) of 100 ms, acoustic filter of 10 to 100 Hz, amplification of 75.000 × .

After recording the responses performed with unilateral and bilateral stimulation, the binaural interaction component could be calculated. In order to obtain the sum of the waves obtained by the right (D) and the left (L) ear stimulation followed by the subtraction of the obtained wave by bilateral stimulation (BI): BIC = ((D + E) - BIN), a second software was used (ASC II) for extracting the waves, which extracted the 256 amplitude points of each wave allowing to achieve grand-average of the records of each group and then the sum and subtraction to obtain the BIC.

AMRL components are generated via the inferior colliculus, medial geniculate body, formation reticularis and primary auditory area along with associated areas and the corpus callosum. The waves were identified based on the consistency of the latency and amplitude values of their components. Thus, the Na component is among the first highest negative peak and lies between 12 and 27 milliseconds (ms); Pa o peak highest positive after the Na wave, between 25 and 40 ms; Nb is the positive peak immediately after Pa, between 30 and 50 ms and Pb, the subsequent higher positive peak, immediately after Nb, between 45 and 65 ms.

Regarding Na-Pa latency and interamplitude, unilateral and bilateral stimulation was in both groups. The peaks in the BIC waveform were labeled as 1^st^ negative peak, 1^st^ positive peak, and 2^nd^ negative peak. To control possible biases, the analysis of records, identification, and marking of each AMLR component was carried out by two judges.

The distribution of normality was verified by the Shapiro-Wilk test with Liliefors correction. An analysis of Repeated Measures Mixed-Design Ancova was performed to observe the group effect, condition, and interaction (group vs. condition) controlling the effect of covariate age. Box's M Test was used to verify if the covariance matrices of the dependent variables were the same for the two groups and the Mauchly's Test was used to test the sphericity hypothesis. In the case of rejection of the sphericity hypothesis, the analyzes were based on the Greenhouse-Geisser multivariate test. The main effect within the group and/or condition was analyzed by the Bonferroni multiple comparison test. The adopted confidence level was 5%.

## Results

The mean age in G1 was 65.75 years, and 67.4 years in G2. The participants' schooling level ranged from incomplete elementary school to complete higher education; the average of the Mini-Mental State Examination (MMSE) was 26.81 points.

Table 1 shows the comparisons among the AMLR components: Na, Pa, Nb, Pb and Na-Pa interamplitude, obtained in the three conditions: unilateral and bilateral stimulation with recording at C3A1 and C4A2 in the elderly without and with hearing loss.

**Table 1:** Mean and standard deviation of the latency values of components Na, Pa, Nb, Pb and Na-Pa interamplitude in the conditions right and left ear stimulation and both ears simultaneously, recorded at C3A1 and C4A2 in G1 and G2.

	Group				Ancova		
	G1 (n = 20)		G2 (n = 20)		p	P	p
Condition	Mean	SD	Mean	SD	Group	Condition	Interaction
RE_C4A2Ipsi_LatNa	18.5	3.9	19.7	3.6	0.970	0.823	0.154
LE_C4A2Contra_LatNa	20.6	4.4	19.3	2.5			
Bi_C4A2_LatNa	19.6	4.7	19.8	3.6			
RE_C3A1Contra_LatNa	19.6	3.4	**22.7^a,b^**	3.0	0.067	0.020**	0.108
LE_C3A1Ipsi_LatNa	18.2	5.1	**19.3^a^**	3.0			
Bi_C3A1_LatNa	19.3	3.8	**19.0^b^**	3.2			
RE_C4A2Ipsi_LatPa	31.6	4.4	30.3	3.8	0.437	0.873	0.924
LE_C4A2Contra_LatPa	31.8	4.0	30.9	4.3			
Bi_C4A2_LatPa	32.3	4.9	31.4	4.7			
RE_C3A1Contra_LatPa	29.6	3.1	32.0	4.4	0.576	0.472	0.081
LE_C3A1Ipsi_LatPa	31.0	4.5	31.1	4.6			
Bi_C3A1_LatPa	31.4	4.7	30.1	4.5			
RE_C4A2Ipsi_LatNb	45.0	5.0	45.4	4.4	0.831	0.356	0.763
LE_C4A2Contra_LatNb	47.8	5.1	46.0	11.1			
Bi_C4A2_LatNb	49.2	5.7	49.7	4.3			
RE_C3A1Contra_LatNb	47.2	6.7	45.9	5.9	0.719	0.214	0.348
LE_C3A1Ipsi_LatNb	46.7	6.2	48.2	5.5			
Bi_C3A1_LatNb	50.1	5.4	49.5	3.9			
RE_C4A2Ipsi_LatPb	51.8	18.0	53.5	18.6	0.680	0.442	0.962
LE_C4A2Contra_LatPb	43.3	25.8	47.8	24.8			
Bi_C4A2_LatPb	42.5	28.8	46.3	27.6			
RE_C3A1Contra_LatPb	41.1	27.7	46.8	24.4	0.230	0.169	0.428
LE_C3A1Ipsi_LatPb	43.5	26.2	57.9	14.3			
Bi_C3A1_LatPb	42.5	28.8	45.8	27.5			
RE_C4A2Ipsi_AmpNaPa	-0.90	0.59	-0.65	0.57	0.471	0.070	0.695
LE_C4A2Contra_AmpNaPa	-1.04	0.68	-0.91	0.81			
Bi_C4A2_AmpNaPa	-1.21	0.68	-1.06	0.78			
RE_C3A1Contra_AmpNaPa	**-1.21^a^**	0.68	**-0.73^a^**	0.54	0.156	0.195	0.038*
LE_C3A1Ipsi_AmpNaPa	-0.97	0.56	-0.99	0.83			
Bi_C3A1_AmpNaPa	-1.19	0.58	-0.82	0.48			

Caption: RE, Right Ear; LE, Left Ear; Bi, Bilateral; Ipsi, Ipsilateral; Contra, Contralateral; Lat., Latency; G1, Elderly without hearing loss; G2, Elderly with hearing loss at high frequencies; n, Number of subjects.

Note: * p ≤ 0.05 for significant effect by Ancova; ** p ≤ 0.05 for significant effect by Ancova with a significant effect of covariable age; same superscript letters indicate significant differences by the Bonferroni multiple comparison test.

There was a difference between the elderly with hearing loss in the latency of the Na component when compared to the stimulation of the right and left ears of the record in C3A1 (left hemisphere) with a significant effect for the covariate age so that older elderly people with hearing loss had worse performance.

A difference was also observed between the groups of elderly people with and without loss of Na-Pa amplitude in C3A1 (left hemisphere) when stimulated in the right ear, and the amplitude was lower in elderly people with hearing loss.

Table 2 shows the results of each component, Na, Pa, Nb, Pb and Na-Pa interamplitude compared in each record channel according to the stimulated ear, thus, for each condition (stimulation mode) comparison was performed between the two recording channels C3A1 and C4A2.

**Table 2:** Mean and standard deviation of the components Na, Pa, Nb, Pb and Na-Pa interamplitude obtained with stimulation of the right ear, left ear and both ears simultaneously compared in conditions C3A1 and C4A2 in each group.

	Group				Ancova		
	G1 (n = 20)		G2 (n = 20)		P	p	p
Condition	Mean	SD	Mean	SD	Group	Condition	Interaction
RE_C4A2Ipsi_LatNa	18.45	3.87	19.68	3.63	0.019*	0.986	0.232
RE_C3A1Contra_LatNa	**19.64^a^**	3.38	**22.73^a^**	2.96			
LE_C3A1Ipsi_LatNa	**18.18^a^**	5.13	19.30	2.95	0.885	0.012*	0.017**
LE_C4A2Contra_LatNa	**20.64^a^**	4.43	19.25	2.45			
Bi_C3A1_LatNa	19.25	3.80	18.98	3.24	0.999	0.361	0.592
Bi_C4A2_LatNa	19.62	4.70	19.82	3.59			
RE_C4A2Ipsi_LatPa	**31.62^a^**	4.44	30.33	3.80	0.561	0.601	0.013*
RE_C3A1Contra_LatPa	**29.61^a^**	3.08	32.00	4.40			
LE_C3A1Ipsi_LatPa	30.95	4.47	31.13	4.56	0.933	0.768	0.581
LE_C4A2Contra_LatPa	31.75	3.99	30.92	4.26			
Bi_C3A1_LatPa	31.42	4.71	30.07	4.48	0.509	0.606	0.851
Bi_C4A2_LatPa	32.25	4.95	31.36	4.68			
RE_C4A2Ipsi_LatNb	45.01	5.01	45.45	4.42	0.752	0.216	0.196
RE_C3A1Contra_LatNb	47.17	6.74	45.93	5.94			
LE_C3A1Ipsi_LatNb	46.73	6.20	48.18	5.53	0.942	0.475	0.320
LE_C4A2Contra_LatNb	47.78	5.09	45.96	11.06			
Bi_C3A1_LatNb	50.06	5.42	49.49	3.89	0.635	0.178	0.388
Bi_C4A2_LatNb	49.25	5.67	49.70	4.25			
RE_C4A2Ipsi_LatPb	51.79	17.98	53.49	18.63	0.563	0.475	0.714
RE_C3A1Contra_LatPb	41.14	27.74	46.79	24.39			
LE_C3A1Ipsi_LatPb	43.53	26.15	57.89	14.26	0.127	0.113	0.150
LE_C4A2Contra_LatPb	43.33	25.79	47.76	24.78			
Bi_C3A1_LatPb	42.49	28.76	**45.78^a^**	27.45	0.679	0.014**	0.725
Bi_C4A2_LatPb	42.54	28.84	**46.33^a^**	27.63			
RE_C4A2Ipsi_AmpNaPa	-0.90	0.59	-0.65	0.57	0.047*	0.209	0.184
RE_C3A1Contra_AmpNaPa	-**1.21^a^**	0.68	**-0.73^a^**	0.54			
LE_C3A1Ipsi_AmpNaPa	-0.97	0.56	-0.99	0.83	0.978	0.318	0.570
LE_C4A2Contra_AmpNaPa	-1.04	0.68	-0.91	0.81			
Bi_C3A1_AmpNaPa	-1.19	0.58	-0.82	0.48	0.270	0.804	0.250
Bi_C4A2_AmpNaPa	-1.21	0.68	-1.06	0.78			

Caption: RE, Right Ear; LE, Left Ear; Bi, Bilateral; Ipsi, Ipsilateral; Contra, Contralateral; Lat., Latency; G1, Elderly without hearing loss; G2, Elderly with hearing loss at high frequencies; n-number of subjects.

Note: * p ≤ 0.05 for significant effect by Ancova; ** p ≤ 0.05 for significant effect by Ancova with significant effect of covariable age; same superscript letters indicate significant differences by the Bonferroni multiple comparison test.

Table 2 demonstrates that the latency of Na component in the elderly without hearing loss obtained with the stimulation of the left ear and recorded at C4A2 presents a higher value of latency, with age as an important factor for the increase of this latency.

There was a significant difference between the latency of Pa component obtained with right ear stimulation and recorded at C4A2.

It is also observed that the Pb component obtained through bilateral stimulation and recorded at C4A2 presents a higher latency than that recorded at C3A1, with age as a significant factor influencing the increase in latency.

**Fig. 1:**
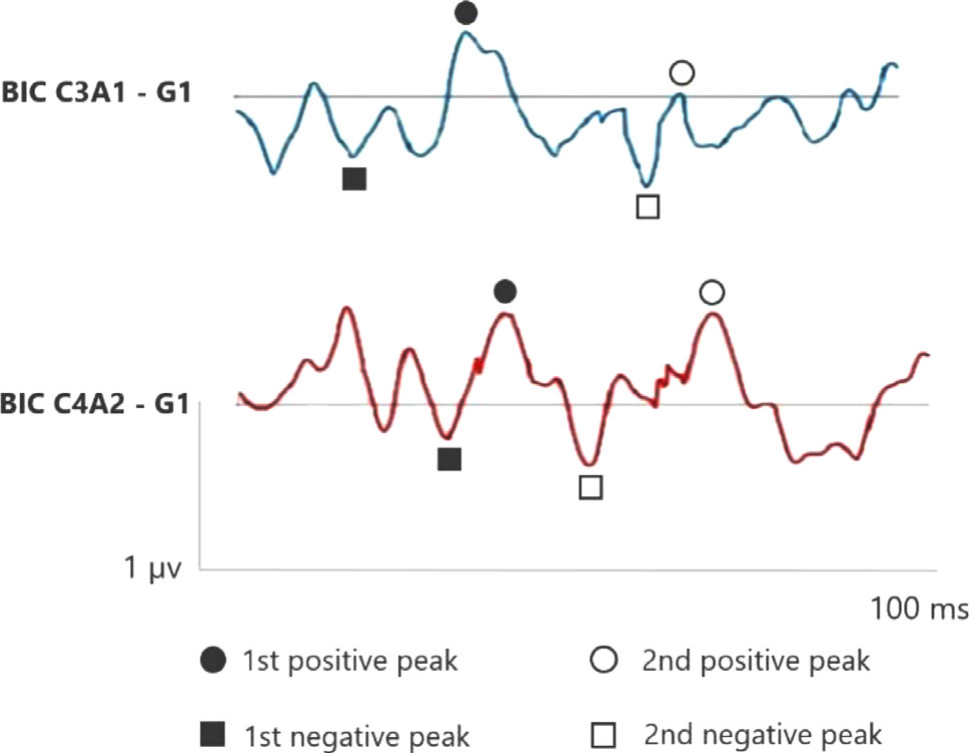
BIC C3A1 and C4A2 in the elderly without hearing loss (G1). BIC, Binaural Interaction Component.

**Fig. 2:**
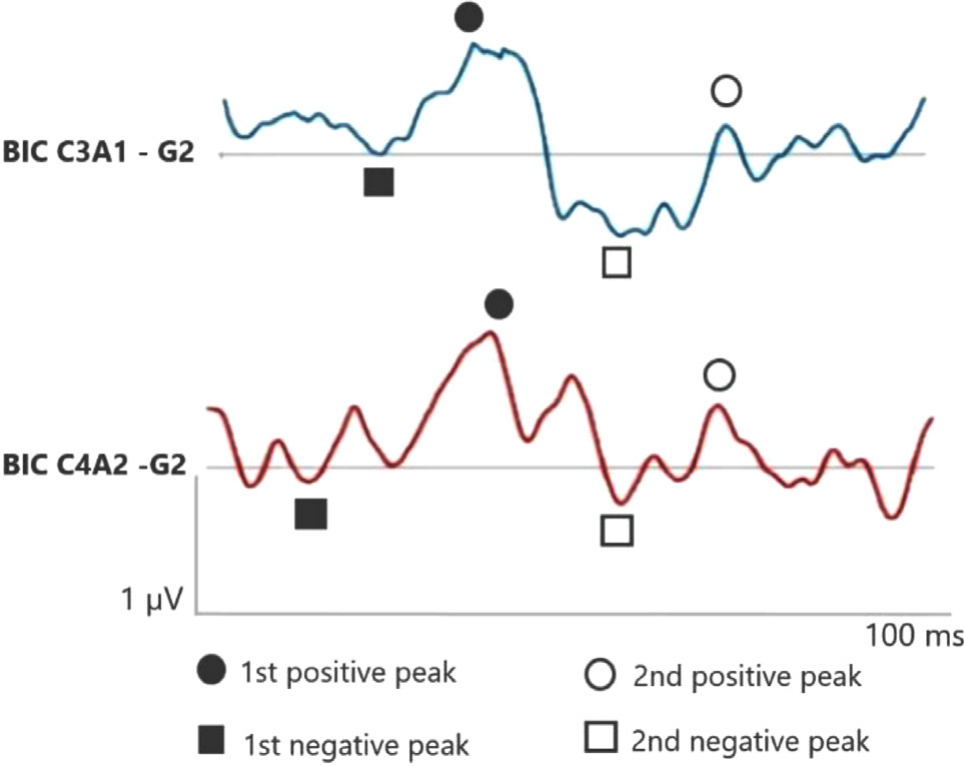
BIC C3A1 and C4A2 in the elderly with hearing loss at high frequencies (G2). BIC, Binaural Interaction Component.

Fig. 1, Fig. 2 correspond to the waves of the BIC performed through ASC II, in both groups (G1 and G2), respectively. Therefore, as two channels were used for carrying out AMLR, BIC was obtained in the two channels: C3A1 and C4A2.

In the elderly without hearing loss, a more prominent 1^st^ negative peak and 1^st^ positive peak inter-amplitude in C3A1 is observed, whereas Na amplitude is more negative than when compared in the elderly with hearing loss.

In addition, it is observed that binaural interaction at C4A2 in the elderly without hearing loss also presents a better wave morphology when compared to the elderly with hearing loss, and the 1^st^ negative peak, 1^st^ positive peak, 2^nd^ negative peak and 2^nd^ positive peak present a higher amplitude in G1. Moreover, the 2^nd^ positive peak presents a higher amplitude in C4A2 in both groups.

## Discussion

The present study aimed to investigate the auditory function of elderly people through middle latency potentials, and to compare unilateral and bilateral stimulation in elderly people with and without hearing loss. The authors demonstrated that the average latency values of components Na, Pa, Nb and Pb were within the normal range according to the age group, as well as the Na-Pa interamplitude, which was lower in the elderly with and without high-frequency hearing loss.^11^

Few studies address the measurement of the AMLR in the elderly. The paper by Moosavi^10^ showed that only the latency of Nb wave was prolonged in the unilateral stimulation and Pa component in the bilateral stimulation, and the amplitude of the wave increased significantly in the elderly without hearing loss. In the study by Lenzi^12^ there was an increase in the latency of the AMLR components in the elderly, a decrease in amplitude, in addition to lower reproducibility and worsening quality of wave tracing, as found by other authors.^13^

When checking the ear effect (Table 1), it is observed that a longer time is required for the transmission of the crossed auditory information to the primary auditory cortex, passing through regions linked to Na component generation, subcortical and thalamic.^14^, ^15^, ^16^ Thus, the degeneration of the structures of the thalamic-cortical pathways due to aging could produce a loss in this transmission.^17^

These findings can be confirmed in the comparison of the Na-Pa amplitude values (Table 1). At C3A1, the lower amplitude is verified when only one ear is stimulated in the elderly with hearing loss at higher frequencies due to the reduction of neuronal activity in the thalamic-cortical pathways in this group of elderly people.^18^ Na-Pa amplitude is higher when there is bilateral stimulation than when only one ear is stimulated, especially in the elderly with hearing loss at high frequencies recorded at C4A2 (Table 1).^19^

As reported by Fonseca and Costa-Ferreira^20^ due to sensory deprivation, even in high frequencies, the elderly may present impaired hearing abilities and the regions involved in auditory processing can be compromised by affecting listening abilities, also evidenced by the deficient findings of AMLR.^21^ In this study, the AMLR was sensitive to studying the central auditory pathway of the elderly both with hearing loss at high frequencies and without hearing loss.^22^^,^^23^

In the hemisphere comparison, electrode effect, Table 2 shows that these results can be justified as the processing of the non-verbal auditory information occurs in the RH and requires greater participation of the corpus callosum for the acoustic signal processing, which is degraded in the elderly.^19^

According to Jerger et al.^13^ both interaural and inter-hemispheric asymmetries, related to the loss of interhemispheric transference efficiency through the corpus callosum, may be related to age even without the presence of hearing loss, but when there is the presence of this asymmetry it may be even more impaired. There is a disadvantage of the right ear in the elderly when non-verbal stimulation occurs, suggesting the association of aging with atrophy of the corpus callosum fibers, with important implications for the effective use of bilateral listening in the elderly.

Na component is generated from the thalamo-cortical connections, and Pa in the medial portion of Heschl's gyrus, responsible for acoustic recognition and discrimination abilities of the auditory cortex.^15^ Greater activation was also observed in the right temporal lobe in comparison with the left in the elderly, as shown in the study by Nakamura et al.^24^ Pb component is the response of the reticular activation system, which presents large binaural properties.^25^

Therefore, as the latency of the described components was higher at C4A2 than at C3A1 in the elderly with normal hearing, it is possible to observe auditory CNS degradation at the cortical level, even in the absence of hearing loss.

In relation to BIC when comparing C3A1 and C4A2, it occurs at the cortical level in both groups, and it is possible to visualize the 2^nd^ positive peak higher at C4A2 in the elderly with and without hearing loss when compared to C3A1. As the 2^nd^ positive peak is generated from the thalamic nuclei, entry of the reticular activation system.^26^ Then, greater activation in this region in both the elderly without hearing loss and those with loss at high frequencies.

However, in this study, there was an increase in the amplitude of the 2^nd^ positive peak in the elderly,^26^ suggesting the occurrence of structural and neurochemical changes and inhibition reduction in the reticular system causing an increase in amplitude in this population. This may reflect diminished cortical and subcortical capacity related to inhibition of response to repetitive auditory stimuli that do not require attention effort.^25^^,^^27^

It was possible to conclude that the AMLR in the ear effect observed in the elderly group with hearing loss shows a slower unilateral auditory information transmission, resulting in losses in the thalamic-cortical transmission and poor quality in the central auditory pathway response with aging in the elderly with hearing loss at high frequencies. When the electrode effect was observed in the elderly without hearing loss, the differences in the responses between the hemispheres can indicate damages to the cortical level even in the absence of hearing loss at high frequencies. Finally, binaural interaction in the cortical and subcortical regions is impaired with aging, with greater impairment in elderly individuals with hearing loss at high frequencies.

Thus, the AMLR showed to be a sensitive examination to investigate neuroauditory disorders in the elderly, especially related to high-frequency hearing loss and primary auditory cortex dysfunctions caused by the aging process. In addition, the BIC from the AMLR reflected damages in the binaural auditory pathways at the cortical level in the elderly, ensuring the integral assessment of the central auditory system and allowing them to choose a more effective form of auditory rehabilitation, especially in this population.

However, conducting behavioral tests that assess central auditory processing associated with electrophysiological measures would broaden the analysis of the central auditory system in the elderly, a methodological point that should be considered in future studies.

## Conflicts of interest

The authors declare no conflicts of interest.
